# The use of supportive communication when responding to older people’s emotional distress in home care – An observational study

**DOI:** 10.1186/s12912-017-0220-8

**Published:** 2017-05-16

**Authors:** Linda Hafskjold, Vibeke Sundling, Sandra van Dulmen, Hilde Eide

**Affiliations:** 1 Faculty of Health and Social Sciences, University College of Southeast Norway, PoBox 7053, N-3007 Drammen, Norway; 2 NIVEL (Netherlands Institute for Health Services Research), Utrecht, The Netherlands; 3 Department of Primary and Community Care, Radboud University Medical Center, Nijmegen, The Netherlands

**Keywords:** Communication, Emotions, Expressed emotion, Health services for the aged, Home care services, Nursing staff, Observational study, Patient comfort

## Abstract

**Background:**

Responding to older people’s distress by acknowledging or encouraging further discussion of emotions is central to supportive, person-centred communication, and may enhance home care outcomes and thereby promote healthy aging. This observational study describes nursing staff’s responses to older people’s emotional distress, and identify factors that encourage further emotional disclosure.

**Methods:**

Audio-recorded home care visits in Norway (*n* = 196), including 48 older people and 33 nursing staff, were analysed with the Verona Coding Definitions of Emotional Sequences, identifying expressions of emotional distress and subsequent provider responses. The inter-rater reliability (two coders), Cohen’s kappa, was >0.6. Sum categories of emotional distress were constructed: a) verbal and non-verbal expressions referring to emotion, b) references to unpleasant states/circumstances, and c) contextual hints of emotion. A binary variable was constructed based on the VR response codes, differentiating between emotion-focused responses and responses that distanced emotion. Fisher’s exact test was used to analyse group differences and determined variables included in a multivariate logistic regression analysis to identify factors promoting emotion-focused responses.

**Results:**

Older people’s expressions of emotional distress (*n* = 635) comprised 63 explicit concerns and 572 cues. Forty-eight per cent of nursing staff responses (*n* = 638) were emotion-focused. Emotion-focused responses were observed more frequently when nursing staff elicited the expression of emotional distress from the patients (54%) than when patients expressed their emotional distress on their own initiative (39%). Expressions with reference to emotion most often received emotion-focused responses (60%), whereas references to unpleasant states or circumstances and contextual hints of emotion most often received non-emotion-focused responses (59%). In a multivariate logistic model, nursing staff’s elicitation of the emotional expression (vs patients initiating it) and patients’ expression with a reference to an emotion (vs reference to unpleasant states or contextual hints) were both explanatory variables for emotion-focused responses.

**Conclusions:**

Emotion-focused responses were promoted when nursing staff elicited the emotional expression, and when the patient expression referred to an emotion. Staff responded most often by acknowledging the distress and using moderately person-centred supportive communication. More research is needed to establish generalizability of the findings and whether older people deem such responses supportive.

## Background

The need for high-quality home care for older people will increase in most of Europe and other Western countries as people live longer [1, 2]. During home care visits, nurses are supposed to provide both instrumental and emotional support. Emotional support can prevent cognitive decline at older ages and maintain older people’s functioning in daily life [3, 4]. Nursing staff play a significant role as part of the older person’s social network [3], and older people’s experience of connectedness with others in their network is closely linked to higher ratings of health and protection against depression [5, 6].

Providing emotional support by responding to patients’ verbal expressions of negative emotions with statements that allow for or explicitly encourage further discussion of emotions in doctor–patient settings has been found to elicit clinically important information, in addition to fostering the doctor–patient relationship [7]. This is assumed to be transferable to other care relationships, such as in nursing. Therefore, it is important to explore how nursing staff engage in communication that acknowledges emotions, facilitates emotional support and comfort [8] and fosters the experience of social connectedness for older people [3]. Research also indicates that nursing staff may influence the care recipient’s positive and negative emotions depending on the parties’ responses to one another [9].

Studies describing communication about emotional distress in home care show that nursing staff in home care can experience communicative challenges, especially when faced with existential issues, fragility and worries from older people during a care situation [10]. Studies also indicate a need for nurses to address topics not just relating to nursing or therapeutic issues [11–13]. In an observational study of Swedish home care visits, older people communicated emotional distress to nursing staff in about half of the visits [14]. Older people’s expressions of emotional distress communicated during home care visits have been described as relating to four main categories: a) worries about relationships with others, b) worries about healthcare-related issues, c) worries about aging and bodily impairment and d) life narratives and value issues [11]. These main categories coincide well with the important features of successful aging at home for frail older people as emphasized in another qualitative study: balancing daily rituals with social engagement in the face of loss and change, in order to retain both capacity and quality of life [12]. Moreover, the need for someone to keep an eye on them and having someone to talk to were described as central to older people receiving home care. An analysis of Norwegian home care visits by Hafskjold, Eide, Holmstrom, Sundling, van Dulmen and Eide [11] revealed the complexity of underlying emotions in older people’s expressions of personal worries in communication with nursing assistants. Further, a Dutch observational study exploring communication with older people found that their level of socio-emotional interaction may be higher than previously reported, and that nurses provide more affective communication (support, concern and empathy) in home care compared with nurses in institutional care [13]. However, Caris-Verhallen, Kerkstra, van der Heijden and Bensing [13] concluded that nurses in home care communicate more about nursing and therapeutic topics, and less about topics labelled as “feelings” (understood as equivalent to the term “emotions” used in this study) and lifestyle related.

### Supportive communication and comforting strategies

The present study builds on the understanding that sensitivity to patients’ needs is linked to the ability to identify and ease suffering [15]. Responses from nursing staff that allow and explore patient expressions of negative emotions are important in order to build trust in the relationship, show emotional support and discover clinical information relevant to the planning of care [8, 16].

Morse, Bottorff, Anderson, O’Brien and Solberg [8] present a communication model that emphasizes the actual process of engaging in patients’ experiences in order to provide comforting responses to their suffering or emotional distress. Here, the ability to perceive the other person’s emotions is not primarily a cognitive process, but rather related to being present and attentive towards the other. Through this process, the nurse may experience emotional empathy, described as empathic insight. Empathic insight enables the nurse to respond in a way that is naturally comforting and supportive to the patient.

Burleson [17] emphasizes how comforting strategies should lead the distressed person to feel better in their immediate situation, but also position them to cope better with distressful events in the future. Supportive communication is defined by Burleson and Macgregor [18] as “the verbal and nonverbal behaviour produced with the intention of providing assistance to others perceived as needing that aid” (p. 374). Further, they state that supporting others is a fundamental form of human interaction, often aiming to relieve the other person’s distress. This is transferable to a care context in the sense that the act of caring includes support and comfort, and care outcomes are related to both easing suffering and enabling coping by the patient [8, 19]. Burleson [17] upholds that messages (comforting strategies) that legitimize and acknowledge the other’s emotions and perspective are more person centred and have a more supportive effect on the recipient compared with messages that deny the other’s emotions and perspective (criticizing, challenging or telling them what to feel). This is similar to how person-centred communication is described in literature relevant for nursing and different clinical settings [20–22].

Burleson developed the Hierarchical Coding System for Sensitivity of Comforting Strategies (HCSSCS) [17, 23] and the method was later adapted to evaluate the degree of empathic accuracy of nurses’ responses to patients’ expressions of emotional distress during consultations in a pain clinic [16]. The nurses’ responses were categorized into one of three levels of comforting strategies: 1) denial of the person’s perspective, 2) implicit recognition or approval of the person’s perspective and 3) explicit recognition of the expressed emotion. The response from the nurses was termed an “empathic accurate response” if the response as a minimum implicitly recognized the patient perspective, and level of accuracy was based on the degree of congruence between the distress expressed by the patient and the verbal and/or non-verbal responses from the nurses [16]. Empathic accuracy can be described as the ability to infer successfully other people’s thoughts and emotions [24]. The extent to which the nurse’s response acknowledges and elaborates on the expressed emotions is at the core of evaluating the response as reflecting a shared perspective with the patient and as being empathically accurate [16].

There is evidence suggesting that successful empathic accuracy is most strongly related to understanding the information extracted firstly from the verbal channel (the words used) and secondly from non-verbal vocal cues (tone of voice, intonation, pauses, etc.), and to a lesser degree the non-verbal channel (body posture, gestures, eye contact, etc.) [24–26]. This supports the assumption that communicating verbally about emotional distress is necessary in order for nursing staff to perceive accurately the other person’s perspective. Sharing and acknowledging emotions can help strengthen the therapeutic bond between an older person and nursing staff if the staff respond in a way that builds trust and understanding [10, 20].

Supportive communication that acknowledges and legitimizes negative emotions is seen as an effective way of addressing verbally expressed worries and distress [17]. The literature does not seem to explore in detail how nursing staff respond to and support emotional distress communicated by older people in home care visits. Therefore, the over-arching aim of this study is to identify the important features of such nurse–patient communication in home care that allow the emotional distress of older person to be shared and discussed. The sub-aims are 1) to describe how nursing staff respond to older people’s expressed worries, and 2) to identify conditions that encourage older people to open up for further disclosure of their emotions.

## Methods

### Design and setting

The study had a descriptive observational design and was part of a large international research project on person-centred communication in home care (COMHOME) [27]. This study reports the Norwegian part of the COMHOME project.

Data were collected from four home care units: three units located in a city of approximately 65 000 residents and one unit in a rural municipality of approximately 5000 residents. The encounters took place in the private homes of the older people. Real-time communication unfolding between the older person and the registered nurse (RN) or nursing assistant (NA) during the visits was audio-recorded and analysed. The units of analysis were the older person’s expressions of emotional distress; concerns, and cues or non-verbal vocal cues, identified by the Verona Coding Definition of Emotional Sequences (VR-CoDES), and the subsequent responses of the RN or NA [28].

### Sample

The study population comprised older people (≥65 years) receiving home care in Norway, and the RNs and NAs providing home care services. In Norway, RNs must have at least a bachelor degree, and NAs must have completed formal education to at least upper secondary school level plus vocational training in nursing.

#### The nursing staff sample

The managers of the home care units recruited in total 33 nursing staff, 16 RNs and 17 NAs. All had received oral and written information about the study from the research group before recruitment. The inclusion criteria were being an RN or an NA providing home care in private homes (not apartments located in nursing homes) with a permanent position. The target sample of nursing staff was 10 participants for each unit, aiming for an equal distribution of RNs and NAs. There are more female nursing staff in the population; therefore, no measures were taken to ensure equal distribution of genders in the sample, besides ensuring that both genders were represented among both RNs and NAs. Regions 1 and 4 organized home care in two units with allocated personnel. Therefore, only seven and six staff members were included, respectively, from regions 1 and 4. The work experience of participating nursing staff ranged from 5 months to 45 years, with an average experience of 17 years (SD, 11; missing data for one NA). Information about age and gender is shown in Table 1.

**Table 1:** Sample of older people, nursing staff and visits

Participants	Women/men	Average age (SD)	Age range
Older people (*n* = 48)	37/11	84 (8)	65–94
Total nursing staff (*n* = 33)	27/6	42 (11)	23–59
Nurse assistants (*n* = 17)	15/2	44 (10)^a^	24–59
Registered nurses (*n* = 16)	12/4	40 (11)^a^	23–56
Visits	Average length of visits in minutes (SD)	Range of length of visits in minutes	
Total (*n* = 196)	17 (14)	1–72	
With nurse assistants (*n* = 99)	17 (14)	1–72	
With registered nurses (*n* = 97)	17 (14)	1–70	

^a^Missing data on 2 nurse assistants and 2 registered nurses

#### The sample of older people

In total, 48 older people participated in the study; see Table 1. Average activities of daily living (ADL) scores, the level of assistance needed to perform a range of daily tasks (0 = no assistance needed, 5 = full assistance needed) [29], varied between 0 and 3.7 (SD, 2.1). Hours of home care included in the care plan ranged from 0.3 to 21.5 h per week (average, 5.2; SD, 5.3). The inclusion criteria were being an older person receiving home care and being able to provide informed consent. Older people considered too frail, based on medical records and clinical experience (e.g., severe dementia, cognitive challenges, final-stage cancers), were excluded by the unit manager.

### Data collection procedure

All participating nursing staff took part in the recruitment of older people, after having received proper instructions by the research group. Data were collected during the period of December 2013 to April 2014. The recruitment started a week before the planned start of the data collection. The data collection was organized for the four units consecutively and aimed to be completed within a week.

The audio recordings of entire home care visits (*n* = 196) were collected using a digital audio recorder (H1 Zoom), worn on the upper arm of the participating nursing staff. One hundred and twenty-two visits included 1–2 identified tasks to be completed, 60 visits had 3–4 tasks and 14 visits had five or more tasks. The duration of the visits ranged from 1 to 72 min, with an average duration of 17 min (SD: 14). The participating RN or NA could encounter the same older person in multiple visits, and the older person could encounter different nursing staff in multiple visits. All care providers encountered at least three different patients.

### Coding methodology: Verona Coding Definitions of Emotional Sequences (VR-CoDES)

To identify emotional talk sequences, all visits were coded with the VR-CoDES – Cue and Concern [30, 31], identifying the older person’s emotional distress as cues and concerns; and the VR-CoDES – Provider Response [32, 33], identifying the nursing staff’s responses to emotional distress.

The coders were the first author (LH) and a research assistant. The first author was trained in the use of VR-CoDES by the last author (HE), an experienced coder and one of the founders of the coding system. When there was a need to resolve issues concerning the implementation of the VR-CoDES in a home care setting or disagreements on the interpretation of specific expressions between the two coders, and to establish consensus about the appropriate use of the VR-CoDES, the last author was consulted.

### Older people’s expressions of emotional distress

VR-CoDES defines *concerns* as clear and unambiguous expressions, where the emotion is current or recent and explicitly verbalized. A *cue* is defined as a verbal or non-verbal hint of an underlying unpleasant emotion, but the expression is lacking clarity. Cues are categorized into seven mutually exclusive categories: vague or unspecific words for the emotion (*cue a*), verbal hints of implicit emotions or unpleasant states/circumstances (*cue b*), phrases emphasizing unpleasant cognitive or physical states (*cue c*), expressions of potential importance (*cue d*), repetition of neutral words or phrases (*cue e*), non-verbal expressions or hints of negative emotions (*cue f*) and verbalized references to an emotion that occurred more than a month ago (*cue g*). Further, all concerns and cues were coded either as expressed by the older people themselves on their own initiative (patient expressed, PE) or as elicited (concerns/cues being solicited, explored or facilitated) by the nursing staff (health-care provider elicited, HPE).

The two coders analysed 32% of the recordings independently to reach acceptable inter-rater reliability. The steps taken in this process followed given recommendations [28]. An inter-rater reliability of a Cohen’s kappa above 0.6 (substantial agreement) was considered sufficient for further analysis [34]. The inter-rater reliability was calculated for the following outcome variables: expressions of emotional distress (*n* = 63, Cohen’s kappa = 0.74); concerns (*n* = 15, Cohen’s kappa = 0.68); and whether the older person took the initiative to express the concern/cue, or the expression was elicited by the health-care provider (*n* = 119, Cohen’s kappa = 0.68). Cohen’s kappa was not calculated for specific cue categories. The inter-rater reliability was comparable to that found in other studies using VR-CoDES [35–37]. The coding system is considered to have high ecological validity, capturing concerns that are experienced as real by the patient [38].

#### Categorization of cues and concerns

Three sum categories of expressed emotional distress were computed: expressions of an emotion by referring to it in words, references to unpleasant states/circumstances and contextual hints of emotion. These categories were respectively labelled as *emotional references*, *emotional states/circumstances* and *contextual hints of emotion. Emotional references* (4 VR codes) were expressions with either clear (*concerns* and *cue g*) or vague words (*cue a*) or non-verbal vocal cues (*cue f*) related to a negative emotion. *Cue f* was coded if the patient sighed, whined, moaned, cried or sobbed, as these are considered self-evident vocal expressions (no words used) of a negative emotion, e.g., crying is understood as a reference to sadness. *Emotional states/circumstances* (2 VR codes) were verbal hints of implicit emotions or unpleasant states/circumstances (*cue b*), or phrases emphasizing unpleasant cognitive or physical states (*cue c*). *Contextual hints of emotion* (2 VR codes) were neutral expressions coded as utterances of emotion because of contextual factors or hints, such as how the expression emerged from the narrative background (*cue d*) or was repeated by the patient (*cue e*); see Table 2.

**Table 2:** The distribution of concerns and cues codes and elicitation by VR-CoDES sum categories and nursing staff

VR-CoDES – Concerns and cues sum category					
		VR-CoDES	Nurse assistant (%)	Registered nurse (%)	Total (%)
Emotional references (*n* = 224) Code-related example Concern: “Then I got a bit sad, thinking oh my God, are they that sick” Cue a: “I really don’t like my eyes at the moment” Cue f: (Crying and snivelling) Cue g: “That was ghastly” (disgust, more than a month ago)	Nursing staff elicited*	Concern	24 (35)	21 (33)	45 (34)
		Cue a	41 (59)	29 (46)	70 (53)
		Cue f^a^	–	–	–
		Cue g	4 (6)	13 (21)	17 (13)
		Sum	69 (100)	63 (100)	132 (100)
	Patient elicited**	Concern	5 (9)	13 (35)	18 (20)
		Cuea	27 (49)	11 (30)	38 (41)
		Cue f	23 (42)	12 (32)	35 (38)
		Cue g	0 (0)	1 (3)	1 (1)
		Sum	55 (100)	37 (100)	92 (100)
Emotional states/circumstances (*n* = 396) Code-related example Cue b: “yes, because everything is just dry” Cue c: “I have no memory either, that is the worst”	Nursing staff elicited	Cue b	125 (97)	92 (99)	217 (98)
		Cue c	4 (3)	1 (1)	5 (2)
		Sum	129 (100)	93 (100)	222 (100)
	Patient elicited	Cue b	90 (97)	81 (100)	171 (98)
		Cue c	3 (3)	0 (0)	3 (2)
		Sum	93 (100)	81 (100)	174 (100)
Contextual hints to emotion (*n* = 15) Code-related example Cue d: “I have a tumour in the stomach” (talking about food) Cue e: “It still resides a bit” (third time repetition, about symptoms after stroke)	Nursing staff elicited	Cue d	1 (100)	0 (0)	1 (50)
		Cue e	0 (0)	1 (100)	1 (50)
		Sum	1 (100)	1 (100)	2 (100)
	Patient elicited	Cue d	3 (27)	0 (0)	3 (23)
		Cue e	8 (73)	2 (100)	10 (77)
		Sum	11 (100)	2 (100)	13 (100)
		Total	358 (56)	277 (44)	635 (100)

“..” = Examples of older people’s expressions and designated concern/cue code found in the material

*Fisher’s Exact Test: *p* = 0,036 between type of expression with emotional reference expressed to either nurse assistant or registered nurse

**Fisher’s Exact Test: *p* = 0,005 between type of expression with emotional reference expressed to either nurse assistant or registered nurse

^a^not applicable (only patient elicited)

### Nursing staff responses

VR-CoDES – Provider Response codes the nursing staff response immediately following a concern or cue [33]. The coding has two dimensions. First, the coder identifies whether the response refers to the concern/cue *explicitly* or *not explicitly*, e.g., maintaining wording or key elements of the concern/cue or not. Second, the coder determines whether the response performs the function of *providing space* or *reducing space* for further disclosure of the concern/cue, e.g., allowing the patient to talk more about their expressed emotional distress or not. The coding system includes 17 mutually exclusive response codes. Application of the system to audio recordings does not permit use of the code “silence – non-explicit providing space”, and by default the code “ignore – non-explicitly” is used. The code “postponing – reducing space” was not identified in our data. Therefore, these two codes are not included in the sum categories for provider responses (Table 3).

**Table 3:** Frequency of response categories defined by communicative function as described in VR-CoDES- Provider Response

Emotion-focused responses (*n* = 304)		Content-focused responses (*n* = 203)		Ignoring or blocking responses (*n* = 131)	
	n (%)		n (%)		n (%)
Back-channel N-E, PS “Mm”	107 (35)	Information-advise N-E, RS “I will just put on this here, and wrap this around”	75 (37)	Ignoring NE, RS (Overlook the concern/cue)	114 (87)
Acknowledgement N-E, PS “Right”	100 (33)	Content exploration E, PS “Oh, no, did it break just outside, here?”	51 (25)	Shutting down N-E, RS “Well then”	16 (12)
Implicit Empathy N-E, PS “I understand”	36 (12)	Information-advise E, RS “Oh, no, this job is not so bad”	42 (21)	Active blocking E, RS “I will not comment on that, but I don’t experience it as being that bad”	1 (1)
Active invitation N-E, PS “How do you mean?”	35 (12)	Content acknowledgement E, PS “Yes, so it was you who had to clean up afterwards, yes”	29 (14)		
Affective acknowledgement E, PS “That is troubling”	13 (4)	Switching response E, RS “Yes, we can, and that will be no extra for us because we also visit your neighbour”	6 (3)		
Affective exploration E, PS “You don’t like them?”	10 (3)				
Empathic response E, PS “Of cause you miss her, I can understand that”	3 (1)				

*N-E* Non-Explicitly referred to the concern/cue

*E* Explicitly referred to the concern/cue

*RS* Reduce Space for further disclosure of the concern/cue

*PS* Provide Space for further disclosure of the concern/cue [30]

“..” Examples of responses found in the material

Cohen’s kappa was calculated for coder agreement for the response codes that differentiated between providing space and reducing space (and also those differentiating between explicit and non-explicit references to the concern/cue, as these codes occurred within the providing/reducing space dimension) (*n* = 31, Cohen’s kappa = 0.75). Cohen’s kappa was not calculated for the individual response codes.

#### Categorization of provider responses

The categorization of the response codes (Table 3) was based on the function assigned to each response as described in the VR-CoDES manual; that is, whether the response showed that the health-care provider had noticed the patient’s expressed emotion and focused on the content of the patient’s expression, or ignored or blocked the expression [33].

#### Emotion-focused responses (7 VR response codes)

All seven codes in this category indicate that the response functioned to provide space for emotional expression, either explicitly or non-explicitly. The nursing staff notice the emotion and provide a response allowing further disclosure of the emotion. Non-explicit responses (no explicit reference to the previous concern/cue), include back channels, acknowledgement, an active invitation to speak further and implicit empathy and, explicit responses (specifically mentions either the content/topic or the emotion in the previous concern/cue) include acknowledging or exploring the emotion or providing explicit empathy.

#### Content-focused responses (5 VR response codes)

Two response codes in this category focus explicitly on the content of the cue or concern and provide space for, acknowledge or explore the content of the cue or concern. Two response codes explicitly refer to the concern/cue but reduce space for further disclosure by switching (e.g., refers the patient to a third party/agency to talk to them about the concern/cue) or giving advice and specific information. One response code reduces space for further disclosure by providing non-specific information or advice.

#### Ignoring or blocking responses (3 VR response codes)

These codes include responses that seem to ignore the concern/cue completely, non-explicitly diverge from the concern/cue or actively block the concern/cue by refusing to talk more about the topic and devaluing what is said by the patient.

### Statistical analysis

Statistical analysis was performed with IBM SPSS Statistics, version 24.0 (IBM Corp, New York, USA). The dataset was checked for missing data. Variables describing the older people had no missing data.

The outcome variable for the study was whether nursing staff responses opened space for further disclosure of emotion (emotion-focused responses). A binary variable was computed, differentiating between “emotion-focused responses” and “responses focusing on content or responses ignoring or blocking the concern/cue”. The data were fitted to a logistic model to identify predictors of emotion-focused responses.

Possible explanatory variables of the nursing staff responses, such as characteristics of the care provider and patient, the individual visit, and the type and expression of concerns/cues, were explored using frequency and summation tables. Percentages were rounded to whole numbers. Group differences were analysed using Fisher’s exact tests and the significance level was set at <5%. To identify explanatory variables for nursing staff responses, variables with a significance level of ≤25% in univariate logistic regression were included in the multivariate logistic regression analysis [39]. In crosstab analysis, available data were analysed, whereas in univariate logistic regression, the default solution in SPSS of listwise deletion was used.

In order to explore patient expressions of emotion (verbal or non-verbal) versus less direct suggestions of emotion, a binary variable capturing this distinction was computed. This variable differentiated concerns and cues that referred to an emotion (i.e., *emotional references*) from cues referring to states/circumstances or contextual hints of emotions (i.e., *emotional states/circumstances* and *contextual hints of emotions*). This nominal binary variable allowed exploration of whether linguistic properties of the patient’s communication influenced the nursing staff’s responses, and thus was important for answering our research questions. This also strengthened statistical power by combining two categories with limited numbers of observations: *emotional states* and *contextual hints of emotion* (Table 2). The binary variable was used in the logistic regression analysis.

### Ethical considerations

This study has been conducted in compliance with the World Medical Association Declaration of Helsinki: ethical principles for medical research involving human subjects [40].

Local newspapers informed the public about the study in advance of data collection, to inform the community about why care providers would be wearing audio recorders. Nursing staff who were well known to the older people receiving home care provided verbal and written information about the study, before asking for written consent. All participating older people received a telephone call from the unit manager after the audio-recorded visits, and were given the opportunity to withdraw from the study. If the patient had problems answering the phone, a member of staff who had not been part of the visits addressed this in person. None of the patients used the opportunity to withdraw.

## Results

### Concerns and cues, and how they were elicited

We identified 635 expressions of emotional distress, of which 63 (10%) were concerns and 572 (90%) were cues. In all, 224 (35%) concerns/cues were *emotional references*, 396 (62%) were *emotional states/circumstances* and 15 (2%) were *contextual hints of emotion* (Table 2).

In 56% of the cases, the health-care provider elicited the concerns/cues. Within the *emotional references* category, there were differences between the types of concerns/cues patients expressed depending on whether the provider was an RN or an NA; see Table 2. Clearly verbalized emotions (*concerns* and *cue g*) were more frequently expressed to an RN, whereas vague and unspecific words (*cue a*) and non-verbal vocal expressions (*cue f*) were more frequently expressed to an NA. This applied to both concerns/cues expressed by patients on their own initiative and those elicited by nursing staff. In general, the relative frequencies of patient initiation of disclosure and nursing staff elicitation of disclosure did not vary with the nursing staff’s professional background (RN or NA).

### Responses

The coding process identified 638 responses. The additional three VR response codes compared with the total number of concerns and cues are due to three patient expressions being met by responses that represented two different VR response codes (i.e., there were two units of analysis within a single turn of provider talk) [28]. These responses were all to concerns/cues categorized as *emotional references*. In total, 304 (48%) responses opened up the space for further disclosure of the emotion, 203 (32%) were aimed at the content of the concern/cue, 130 responses (20%) ignored the emotional expression and one response (0.2%) blocked the patient. The types of responses in the three response categories, including examples extracted from the material for all eligible response codes, are provided in Table 3.

### Interaction – responding to cues and concerns

There was a significant difference in responses depending on whether the patient spontaneously expressed the concern/cue or this was elicited by the nursing staff. An emotion-focused response was observed more frequently when the concern/cue was elicited by the nursing staff (194 out of 359) than when the concern/cue was spontaneously expressed by the patient (110 out of 279); see Table 4. This pattern did not significantly differ between RNs and NAs. The distribution of emotion- and non-emotion-focused responses is described in Table 4.

**Table 4:** Nursing staff responses to concerns and cues by VR-CoDES sum categories and elicitation

	Emotion focused responses (*n* = 304)		Non-emotion focused responses (*n* = 334)		All responses (*n* = 638)	
	Patient elicited	Nursing staff elicited	Patient elicited	Nursing staff elicited	Patient elicited	Nursing staff elicited
VR-CoDES sum categories	n (%)	n (%)	n (%)	n (%)	n (%)	n (%)
Emotional references	43 (39)	92 (47)	49 (29)	43 (26)	92 (33)	135 (38)
Emotional states/circumstances	59 (54)	101 (52)	115 (68)	121 (73)	174 (62)	222 (62)
Contextual hints to emotion	8 (7)	1 (0)	5 (3)	1 (0)	13 (5)	2 (1)
Total responses	110	194	169	165	279	359

When categorizing concerns/cues using a binary variable differentiating between patient expressions that referred (verbally or non-verbally) to an emotion or not, 60% of the *emotional references* received emotion-focused responses. The patients’ expressions that did not refer to an emotion verbal or non-verbally, i.e., *emotional states/circumstances* and *contextual hints of emotion*, were met with non-emotion-focused responses in 59% of the cases. This pattern was consistent regardless of whether the patient spontaneously expressed the concern/cue or this was elicited by the health-care provider.

In a multivariate logistic regression analysis adjusted for the individual patient and nursing staff, predictors of responses that opened up space for further disclosure of the emotion were when the health-care provider elicited the concern/cue and when the concern/cue included a reference to an emotion, i.e., *emotional references* (Table 5). The Hosmer–Lemeshow goodness-of-fit test showed that the model prediction did not significantly differ from the observed values (*p* = 0.145), supporting model fit [39].

**Table 5:** Parameter estimates for predictors of responses opening up for further disclosure of emotion

	Univariate logistic regression				Multivariate logistic regression			
Independent variables	Intercept	Parameter estimate	Standard error	*P* value	Intercept	Parameter estimate	Standard error	*P* value
Elicitation (binary^a^)	−0,429	0,591	0,162	<0,001	−1,380	0,567	0,166	<0,001
Concerns/cues with or without reference to an emotion (binary^b^)	−0,359	0,743	0,168	<0,001		0,755	0,172	0,001
The patient	−0,686	0,002	0,001	0,008		0,002	0,001	0,02
The nursing staff	−0,323	0,015	0,008	0,078		0,009	0,009	0,33

^a^Nursing staff elicited

^b^Concerns/cues with reference to emotion

## Discussion

This study identifies important features of supportive communication in home care, in which topics causing emotional distress for the older person are acknowledged and the nursing staff invite further disclosure of the emotion. Such behaviour is more likely to occur when the nursing staff elicit the emotional expression and when the patient clearly expresses or hints to an emotion, termed *emotional references* in this paper.

Similar to Eide, Sibbern and Johannessen [16], the present study found that nursing staff mostly use minimal encouragement (yes, ok, mm, etc.) when responding to older people’s emotional distress. However, 20% of nursing staff’s responses were coded as ignoring or blocking the expressed emotional distress; these types of responses can be understood as missing or denying the patient’s perspective, and are therefore described as exhibiting low person-centredness, defined as level 1 by the HCSSCS [16, 17]. The rarity of such responses may indicate that nursing staff working in home care usually recognize emotional distress and acknowledge it as their first response. Such a response pattern has been characterized as empathically accurate [16]. On the other hand, only 8% of the responses explicitly referred to the expressed emotion (affective acknowledgment or exploration, and empathic response), which can be defined as level 3 of the HCSSCS [16, 17]. Level 3 includes explicit recognition of the emotion felt by the patient and elaboration of the patient’s perspective. Given that level 3 responses have been stated to be more effective in actually providing emotional support [17], and emotional support has shown a positive effect on ensuring high-functioning daily life for older people [3], improving health-care providers’ attention and responses to emotional distress could prove valuable in home care for older people.

Paying attention to emotion has been described as an important aspect of providing and receiving social support from the patient’s perspective [12], and as necessary for achieving empathic insight [8]. In this study, using a word describing the emotion or making a non-verbal reference to the emotion can be seen as an empathically accurate response [16]. The current study also found that emotional distress presented as unpleasant emotional states or hints interpreted from contextual cues is more likely to receive responses focusing on content or providing information; in other words, responses that do not pursue or explore the patient’s emotion. This may be linked to how empathic accuracy is mainly informed by the words being used [24, 25]. Elaborating on a patient’s concern/cue by exploring the patient’s feelings, thoughts and beliefs has been described as necessary for ensuring holistic care and demonstrating engagement with the patient [41]. The majority of older people’s expressions of emotional distress do not include a word referring to the emotion but rather refer to underlying unpleasant states or use neutral expressions. This is comparable to the results presented in a Swedish home care context [14]. In light of these results, there may be a need for communication skills training to improve health-care providers’ sensitivity to such indirect expressions of emotion by patients and increase the level of person-centredness when responding. One possibility is training based on the VR-CoDES. This has been trialled in a pilot study (the ZORG intervention) with promising results [42].

Addressing emotional issues is recognized as a salient communication challenge and an important skill for nursing staff providing home care for older people [10, 11, 14, 43]. Nursing staff may see themselves as well informed about worries experienced by the individual patient, given that the care relationships are often are continuous over time, allowing for deep insight into the person’s daily life. In this study, nearly half of the concerns/cues were expressed spontaneously by the patient. Such patient-expressed concerns/cues prompted responses in which nursing staff distanced the emotional content of the concerns/cues. Consequently, nursing staff’s first response when unfamiliar with the content or topic raised by the patient, rather than raised by themselves, may be to explore the content or provide information. It may be plausible that nursing staff explore the emotional component once they are already familiar with the practical or topical content raised by the patient. Topical insight may be a very sensible approach if the issue is unclear or unfamiliar to the nursing staff. On the other hand, if this controls which emotional issues the older person is invited to talk about in depth, this may hinder person-centred care that takes the experience, perspective and values of the person as the point of departure [20]. This may also be seen as a communication in which the nursing staff distance themselves from the patients’ suffering and reduce engagement with their emotional experience. This may lead to less comforting outcomes, as engagement and the sharing of experience are claimed to be necessary to reach empathic insight and provide naturally comforting responses [8]. This could be an interesting issue to explore further through intervention studies testing the level of comfort experienced by older people from different responses to emotional distress.

No evidence linked the pattern of patient initiation versus nursing staff elicitation of concerns/cues to nursing staff education, or demographic characteristics such as age or gender. This may be an indication of RNs’ and NAs’ fulfilment of similar roles relating to supportive communication. Still, caution is needed when interpreting these results, especially when considering gender differences, as the sample included in this study had a limited number of male staff members. In the present study, 56% of the concerns/cues were expressed by staff. This finding is contrary to that in a Swedish study of older people’s expressions of emotional distress [14]. There, 63% of the concerns/cues were elicited by staff. Similarly, a study exploring cancer consultations found that 63% of concerns/cues were elicited by the health-care providers (both physicians and nurses) [37], and for adult patients in a pain clinic, 80% of concerns/cues were elicited by the nurse [36]. The lower rate of health-care provider-elicited concerns/cues in our study may be linked to features of the communication taking place in the home care context. The relatively high proportion of concerns/cues expressed spontaneously by patients found in the present study is likely a feature of the home care setting. The home of the patient and the language used in home care may help level out the asymmetrical care relationship compared with consultations taking place in medical offices with a narrow clinical purpose [44, 45], and could encourage the older person to express their worries on their own initiative more freely. In addition, both the number of concerns/cues expressed in general and the number of concerns/cues expressed spontaneously by patients were higher in the Norwegian material compared with a similar analysis from home care in Sweden [14], which may indicate the need to explore further how culture influences emotional talk.

A further interesting finding of this study is that older people seem to moderate how clearly they state their emotions depending on whether they are talking to an RN or NA. RNs seemed to be confronted with explicit expressions of emotional distress (*concerns* and *cue g*) more often than NAs. For NAs, vague words (*cue a*) and non-verbal vocal cues (*cue f*) were more frequent. A study from cancer care found that nurses communicate less effectively in emotionally charged situations [46], suggesting that emotional explicitness may directly influence communication skills. The difference in how older people express emotional distress depending on nursing staff’s professional backgrounds is based on limited observations, and caution is needed when considering these results. Future studies should explore this further. If the relationship is valid, this could have implications for the focus of communication skills training, and it may be important for nursing staff to be aware of these differences when interacting with older people in home care.

### Strengths and limitations

The VR-CoDES categories were merged into broader theoretically founded sum categories to help resolve statistical power issues that would otherwise affect rarely used codes. This approach was favoured based on the study objectives of allowing the exploration of features previously described as important for promoting supportive communication. In the analysis, the focus remained on the word, phrase or other hints embedded in the patient expression understood as carrying the emotion and guiding the decision to classify a patient expression as a cue or concern. From our point of view, if the patient was crying, sighing or moaning, the emotional component was not hidden but rather expressed. Consequently, *cue f* was included in the category of *emotional references*. This deviates from how Heyn, Finset and Ruland [37] merged concerns/cues in their study. They also focused on finding a measure of explicitness, resulting in a binary variable describing *emotional explicitness.* In their study, *cue f* and *cue b* were classified as hiding emotion. This conceptual understanding of *cue f* may be linked to their understanding of explicitness. *Cue f* clearly does not involve explicit words within the VR-CoDES terminology. In this sense, the definition given by Heyn, Finset and Ruland [37] is more appropriate, but considering the plausibility and common-sense logic of interpreting someone as sad when they cry or sob, we propose that our definition is equally sound. As with Heyn, Finset and Ruland [37], all instances of *cue f* in our study were audibly identified since we used audio recordings and not video.

Further, Heyn, Finset and Ruland [37] coded *cues a*, *c*, *d*, *e* and *g* as more descriptive, compared with the non-descriptive qualities of *cues b* and *f*. The present study, on the other hand, groups the cues based on features extracted from the definitions given by the VR-CoDES manual [31]. Both *cue c* and *cue b* are defined as relating to patients’ descriptions of states/circumstances, rather than expressions describing their emotions as is characteristic of codes like *concerns*, *cue a* or *cue g*. Last, we identified cues that were mainly coded based on an interpretation of contextual factors, such as when a patient introduced issues of potential importance, repeated words or phrases or made the utterance stand out from the narrative background (*cues d* and *e*). Even though both analytical approaches are defensible and yield interesting results, this difference in operationalizing expressed emotion can indicate a need for a deeper conceptual analysis of this issue.

Given the practical considerations taken to ensure a smooth workflow, we could not practise strict strategic inclusion procedures when recruiting older people and nursing staff. This approach allowed us to reach the target value of a minimum of 10 staff members from each unit, a goal stated by management as otherwise impossible to reach. This allowed for strong variation in both the participant characteristics and features of the recorded visits. Further, the choice of self-selection for recruiting nursing staff ensured the ethical principle of voluntary participation was satisfied, but this may have reduced the likelihood of including staff who were less self-confident in their communication skills and, possibly, those less concerned with communication in general. This approach may have increased sampling bias in our material [47].

## Conclusions

Nursing staff give emotion-focused responses to distress in older home care patients primarily when the older person refers to the emotion by name (i.e., using words) or clearly expresses the emotional non-verbally, and when the nursing staff themselves elicit the expression of emotion. The elicitation of and responses to patients’ expressions of emotion are not related to the professional background of nursing staff. Older people in home care seem to spontaneously initiate expressions of emotional distress more often than in traditional medical settings. Nursing staff generally provide an empathically accurate response, but rarely in a highly person-centred way. More research is needed to validate these findings and determine whether this coincides with what older people themselves value as supportive and comforting responses. This study makes a theoretical contribution to our understanding of how nursing staff’s responses to older people’s emotional distress can be evaluated, which provides knowledge for nursing staff and their managers in home care settings and can support communication skills training for these staff.

## Abbreviation

ADL: Activity of daily living
COMHOME: Person-centred communication with older people receiving healthcare
NA: Nurse assistant
RN: Registered nurse
VR-CoDES: Verona coding definitions of emotional sequences
